# Impact of maternally derived antibodies to *Plasmodium falciparum* Schizont Egress Antigen-1 on the endogenous production of anti-*Pf*SEA-1 in offspring

**DOI:** 10.1016/j.vaccine.2019.06.084

**Published:** 2019-08-14

**Authors:** Sangshin Park, Christina E. Nixon, Sunthorn Pond-Tor, Edward R. Kabyemela, Michal Fried, Patrick E. Duffy, Jonathan D. Kurtis, Jennifer F. Friedman

**Affiliations:** a Center for International Health Research, Rhode Island Hospital, The Warren Alpert Medical School of Brown University, Providence, RI 02903, United States; b Department of Pediatrics, The Warren Alpert Medical School of Brown University, Providence, RI 02903, United States; c Graduate School of Urban Public Health, University of Seoul, Seoul 02504, Republic of Korea; d Department of Pathology and Laboratory Medicine, The Warren Alpert Medical School of Brown University, Providence, RI 02903, United States; e Mother Offspring Malaria Studies (MOMS) Project, Seattle Biomedical Research Institute, Seattle, WA 98109, United States; f Muheza Designated District Hospital, Muheza, Tanzania; g Tumaini University, Moshi, Tanzania; h Laboratory of Malaria Immunology and Vaccinology, National Institute of Allergy and Infectious Diseases, NIH, Rockville, MD 20892, United States

**Keywords:** Malaria, Vaccine, Schizont Egress Antigen-1, *Pf*SEA-1, Maternal antibody

## Abstract

**Background:**

We evaluated whether maternally-derived antibodies to a malarial vaccine candidate, *Plasmodium falciparum* Schizont Egress Antigen-1 (*Pf*SEA-1), in cord blood interfered with the development of infant anti-*Pf*SEA-1 antibodies in response to natural exposure.

**Methods:**

We followed 630 Tanzanian infants who were measured their antibodies against *Pf*SEA-1 (aa 810-1023; *Pf*SEA-1A) at birth and 6, 12, 18, and 24 months of age, and examined the changes in anti-*Pf*SEA-1A antibody levels in response to parasitemia, and evaluated whether maternally-derived anti-*Pf*SEA-1A antibodies in cord blood modified infant anti-*Pf*SEA-1A immune responses.

**Results:**

Infants who experienced parasitemia during the first 6 months of life had significantly higher anti-*Pf*SEA-1A antibodies at 6 and 12 months of age compared to uninfected infants. Maternally-derived anti-*Pf*SEA-1A antibodies in cord blood significantly modified this effect during the first 6 months. During this period, infant anti-*Pf*SEA-1A antibody levels were significantly associated with their *P. falciparum* exposure when they were born with low, but not higher, maternally-derived anti-*Pf*SEA-1A antibody levels in cord blood. Nevertheless, during the first 6 months of life, maternally-derived anti-*Pf*SEA-1A antibodies in cord blood did not abrogate the parasitemia driven development of infant anti-*Pf*SEA-1A: parasitemia were significantly correlated with anti-*Pf*SEA-1A antibody levels at 6 months of age in the infants born with low maternally-derived anti-*Pf*SEA-1A antibody levels in cord blood and borderline significantly correlated in those infants born with middle and high levels.

**Conclusions:**

Maternal vaccination with *Pf*SEA-1A is unlikely to interfere with the development of naturally acquired anti-*Pf*SEA-1A immune responses following exposure during infancy.

## 1 Introduction

Malaria remains a leading killer of children globally with an estimated 219 million cases and 435,000 deaths reported in 2017 [1]. Among species of the genus *Plasmodium*, *P. falciparum* is both the most common cause of human malaria infection and the most deadly [1]. The development of a malaria vaccine is considered an important public health goal to prevent malaria morbidity and mortality, nevertheless, vaccine development has focused on a highly restricted repertoire of antigens [2], while vaccine candidates at the latest stages of development confer only modest protection against *P. falciparum* malaria [3], [4].

Clinical *P. falciparum* malaria is uncommon in infants less than 5 months of age [5], [6], [7], [8], [9], [10], and *P. falciparum* infection in infants less than 6 months of age born to mothers with existing immunity in malaria endemic regions is generally found to be of low parasitemia (<100 parasites/μL of blood) [7], [8], [11], [12], [13]. These subclinical infections usually self-resolve, leading to the hypothesis that young infants are equipped with efficient means to control both parasitemia and morbidity [8], [10], [12], [14]. Risk of clinical symptoms increases after 3–6 months of age as demonstrated in studies conducted in sub-Saharan Africa [8], [9], [11], [15], [16]. It is thought that passive transfer of maternal antibody during gestation affords protection from malaria among young infants. Immune responses to a number of antigens have been evaluated in mother-infant pairs in the context of placental malaria or malaria in pregnancy [17], [18], [19], [20], [21], [22]. While several have focused on the impact of placental malaria on malaria outcomes during infancy [2], [3], [4], [6], [23], few studies have investigated the influence of maternal antibodies on infant antibody acquisition.

*P. falciparum* Schizont Egress Antigen-1 (*Pf*SEA-1) has emerged as a promising vaccine candidate antigen. *Pf*SEA-1 is a 244-kDa acidic phosphoprotein expressed in schizont-infected red blood cells and is essential for blood-stage replication. Antibodies against *Pf*SEA-1A arrest parasite egress of late-stage schizont infected erythrocytes in an *in vitro* growth assay. In human studies, antibodies to recombinant *Pf*SEA-1A (aa 801-1023; *Pf*SEA-1A) predict decreased incidence of severe malaria in Tanzanian children and significantly reduced parasite densities in Kenyan adolescents and adults [24]. Moreover, we have recently reported that high maternally-derived anti-*Pf*SEA-1A antibody levels in cord blood, which were highly correlated with anti-*Pf*SEA-1A antibody levels in maternal peripheral blood, significantly reduced the risk of severe malaria in infants up to the age of 12 months [25]. In endemic areas, exposure to parasite antigens results in the development of anti-*Pf*SEA-1A antibodies [24]. Given that anti-*Pf*SEA-1A antibodies protect against severe malaria, we hypothesized that maternally-derived anti-*Pf*SEA-1A antibodies could cross the placenta and interfere with the active production of anti-*Pf*SEA-1A antibodies by the infant. Moreover, studies of several standard childhood immunizations have raised the concern that infant seroconversion could be inhibited by the presence of maternal antibodies [26], [27], [28]. Therefore, understanding whether maternally derived antibodies interfere with early acquisition of immune responses to *Pf*SEA-1 is critical when considering strategies for maternal or infant vaccination [29].

Several studies have observed the dynamics of maternally-transferred and naturally acquired antibodies against *P. falciparum* in infants [30], [31], [32], however, the dynamics of antibody responses to current vaccine candidates, including *Pf*SEA-1, have not been reported. The objectives of this study were to evaluate the role of natural exposure to parasites on anti-*Pf*SEA-1A antibody levels and to examine whether maternally-derived anti-*Pf*SEA-1A antibodies in cord blood modify or interfere with the development of infant anti-*Pf*SEA-1A immune responses following natural exposure to *P. falciparum*.

## 2 Materials and methods

### 2.1 Study participants

The study participants were enrolled in a birth cohort known as the Mother-Offspring Malaria Study (MOMS) Project. Details of the MOMS project have been described in other publications [24], [25], [33], [34]. Briefly, this project was carried out in the Muheza district of Tanzanian, a region with high malaria transmission, from September 2002 through October 2005. The participants consisted of pregnant women, delivered at Muheza Designated District Hospital, and their children born within the study period. Children were followed from birth until 4 years of age. Blood sample collections were routinely performed every 6 months. Children were examined for *P. falciparum* infection using blood smear analysis at birth, biweekly during infancy, monthly after infancy, and any time they were sick. A total of 785 children were followed for up to 3.5 years from birth. Our study population included only infants and children whose cord blood anti-*Pf*SEA-1A levels were measured and whose peripheral anti-*Pf*SEA-1A levels were measured at least once before 24 months of age.

Of the 785 children enrolled, we excluded 155 children whose anti-*Pf*SEA-1A antibody levels in cord blood were not captured or who were not observed at least once after birth, resulting in an analytic sample of 630 children. These subjects provided 1225 infant or child anti-*Pf*SEA-1A antibody measurements and 2357 parasite density examinations.

This study was approved by the Institutional Review Boards of Rhode Island Hospital and Medical Research Coordinating Committee of the National Institute for Medical Research, Tanzania. Written informed consent was obtained from each child’s mother before participation for herself and her newborn.

### 2.2 Sample collection, processing and anti-*Pf*SEA-1 assays

Blood samples were collected by venipuncture and plasma was stored at −80 °C after centrifugation. Parasitemia and parasite density [asexual stage parasites/200 white blood cells (WBCs)] for *P. falciparum* were determined by a Giemsa-stained blood smear prepared from venous or capillary blood. Anti-*Pf*SEA-1A antibody levels were measured using a fluorescent bead-based assay as previously described [34]. Briefly, 100 µg of r*Pf*SEA-1A or 100 µg of Bovine Serum Albumin (BSA) was conjugated to 1.25 × 10^7^microspheres (Luminex) and were pooled and lyophilized in single use aliquots. Reconstituted beads were incubated for 30 min at 37 °C with human plasma samples at 1:80 dilution in Assay Buffer E (ABE, Phosphate Buffered Saline pH 7.4 containing 0.1% BSA, 0.05% Tween-20, and 0.05% sodium azide) in microtiter filter bottom plates (Millipore). Beads were washed three times in ABE by vacuum filtration and incubated for 30 min at 37 °C with biotinylated anti-human immunoglobulin G (IgG) (Pharmingen) diluted 1:1000 in ABE. Beads were washed three times in ABE by vacuum filtration and incubated for 10 min at 37 °C with phycoerythrin conjugated streptavidin (Pharmingen) diluted 1:500 in ABE. Beads were washed three times in ABE by vacuum filtration, re-suspended in ABE and analyzed on a BioPlex 200 multi-analyte analyzer. Fluorescence values for BSA beads were subtracted from r*Pf*SEA-1A beads (unit: relative fluorescence unit, RFU).

### 2.3 Statistical analyses

The outcome of interest was anti-*Pf*SEA-1A antibody levels in infants and young children. Predictors of interest were maternal anti-*Pf*SEA-1A antibody levels measured in cord blood, and parasitemia indicating exposure to natural infection. Parasitemia was defined by average number of parasites on samples enumerated in each time frame. For statistical analysis, we constructed three additional predictors: tertiles of maternal anti-*Pf*SEA-1A antibody levels, a binary parasitemia predictor (presence/absence) in the first 6 months of life, and tertiles of parasitemia during the preceding 6-month window, captured for each distinct window at 6, 12, 18, and 24 months after birth. We then examined the relationships between each of these predictors and infant and young child anti-*Pf*SEA-1A antibody levels using two-way repeated measures analysis of variance.

To examine whether maternally-derived anti-*Pf*SEA-1A antibody levels in cord blood modified infant immune responses, we tested interactions between tertiles of maternally-derived anti-*Pf*SEA-1A antibody levels in cord blood and parasitemia at 6 and 12 months of age, and then performed Spearman correlation analysis between parasitemia and infant anti-*Pf*SEA-1A antibody levels in each maternally-derived antibody tertile. In addition, we performed mediation analysis to examine whether maternally-derived anti-*Pf*SEA-1A antibody levels in cord blood interfered with the development of infant anti-*Pf*SEA-1A antibodies in response to parasitemia according to tertiles of maternally-derived anti-*Pf*SEA-1A antibody levels in cord blood at 6 months of age.

Anti-*Pf*SEA-1A antibody levels and parasitemia were skewed with a long right tail and therefore were natural log-transformed after adding integer 1 to each value for statistical analysis. We performed mediation analysis using Mplus 8 statistical software (Muthén and Muthén, 1998–2017). We performed all other statistical analyses using SAS 9.4 (SAS Institute, Cary, NC). *P* values < 0.05 were considered statistically significant.

## 3 Results

**Fig. 1:**
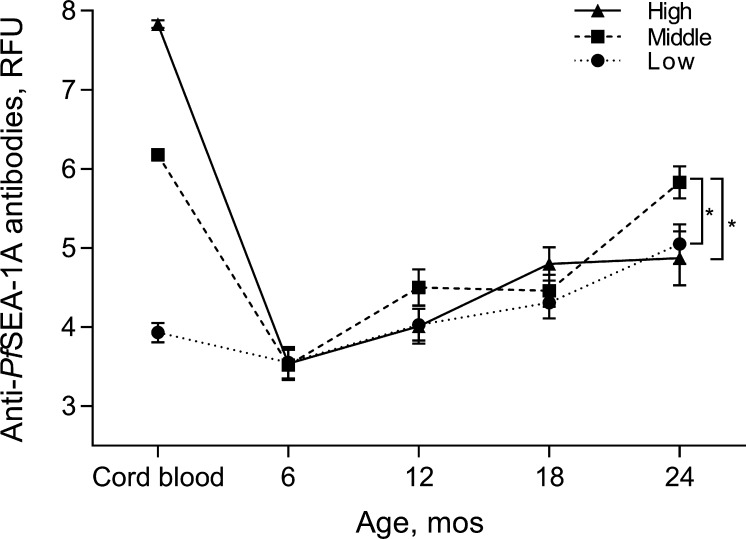
Effects of maternally-derived anti-*Pf*SEA-1A antibodies in cord blood on endogenous anti-*Pf*SEA-1A antibodies measured in infants. Infants were grouped by tertiles of anti-*Pf*SEA-1A antibodies in cord blood. Anti-*Pf*SEA-1A antibody levels were natural log-transformed. Error bars represent 95% confidence intervals. ^*^P < 0.01.

Potential confounders were not significantly different across the tertiles of maternally-derived anti-*Pf*SEA-1A antibody levels in cord blood (Table 1). As expected, infant anti-*Pf*SEA-1A antibody levels decreased during the first 6 months of life and then gradually increased and reached a peak at 24 months (Fig. 1). Prior to 24 months of age, peripheral anti-*Pf*SEA-1A antibody levels did not differ across the tertiles of maternally-derived anti-*Pf*SEA-1A antibody levels in cord blood. At 24 months of age, children born with the middle tertile of maternally-derived anti-*Pf*SEA-1A antibodies had significantly higher anti-*Pf*SEA-1A antibody levels than those with low (*P* value = 0.007) and high (*P* value = 0.003) tertiles.

**Table 1:** Characteristic of the study participants.

	Levels of anti-*Pf*SEA-1A antibodies in cord blood			
Variable	Low (N = 210)	Middle (N = 210)	High (N = 210)	*P* value†
Range of anti-*Pf*SEA-1A antibodies in cord blood, fluorescence units	0–256	257–919	920–26,389	
Maternal characteristics				
Parity, %				
Primigravid	21.4	29.5	27.1	0.23
Secundigravid	21.9	24.3	21.0	
Multigravid	56.7	46.2	51.9	
Children characteristics				
Male, %	50.5	51.0	47.1	0.70
Transmission season at birth, %				
High	48.6	41.9	49.5	0.23
Low	51.4	58.1	50.5	
Hemoglobin genotype, %				
AA	83.8	86.7	81.0	0.28
AS or SS	16.2	13.3	19.0	
Parasitemia (asexual stage parasites/200 WBCs)*				0.73
Between 0 and 6 months	8.1 (3.6–12.6)	11.5 (3.0–20.1)	11.2 (5.5–17.0)	0.96
Between 6 and 12 months	34.3 (19.4–49.2)	45.0 (26.7–63.3)	44.0 (14.2–73.8)	0.62
Between 12 and 18 months	46.6 (23.7–69.5)	57.9 (33.2–82.6)	69.6 (38.2–101.0)	0.29
Between 18 and 24 months	32.0 (13.5–50.6)	66.6 (37.1–96.2)	42.1 (18.3–65.9)	0.16
Subjects with antibody measurement, N (%)				
Birth	210 (100)	210 (100)	210 (100)	NA
6 months	134 (63.8)	145 (69.0)	142 (67.6)	
12 months	116 (55.2)	100 (47.6)	101 (48.1)	
18 months	98 (46.7)	94 (44.8)	85 (40.5)	
24 months	73 (34.8)	78 (37.1)	59 (28.1)	

NA: not applicable.

* Means (95% confidence intervals).

† Tested by Chi-square or two-way repeated measures analysis of variance.

**Fig. 2:**
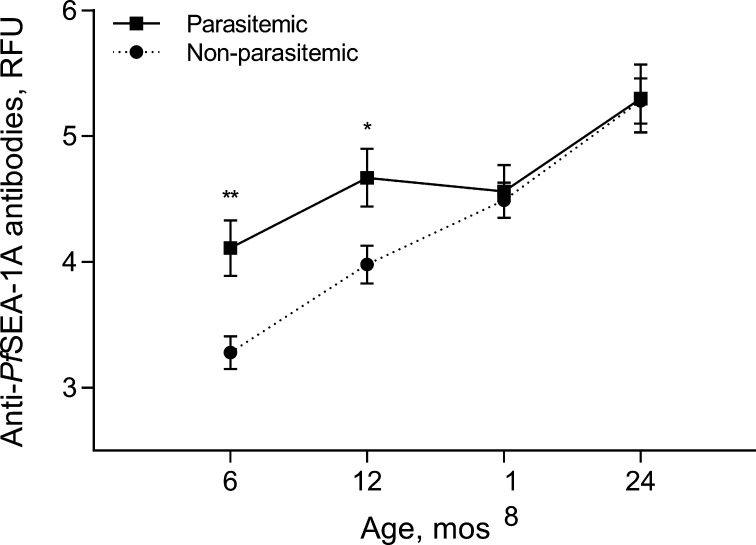
Effects of *P. falciparum* parasitemia experienced from birth to six months of age on anti-*Pf*SEA-1A antibody levels. Anti-*Pf*SEA-1A antibody levels were natural log-transformed. Error bars represent 95% confidence intervals. ^*^P < 0.05 and ^**^P < 0.001 compared with non-parasitemic.

**Fig. 3:**
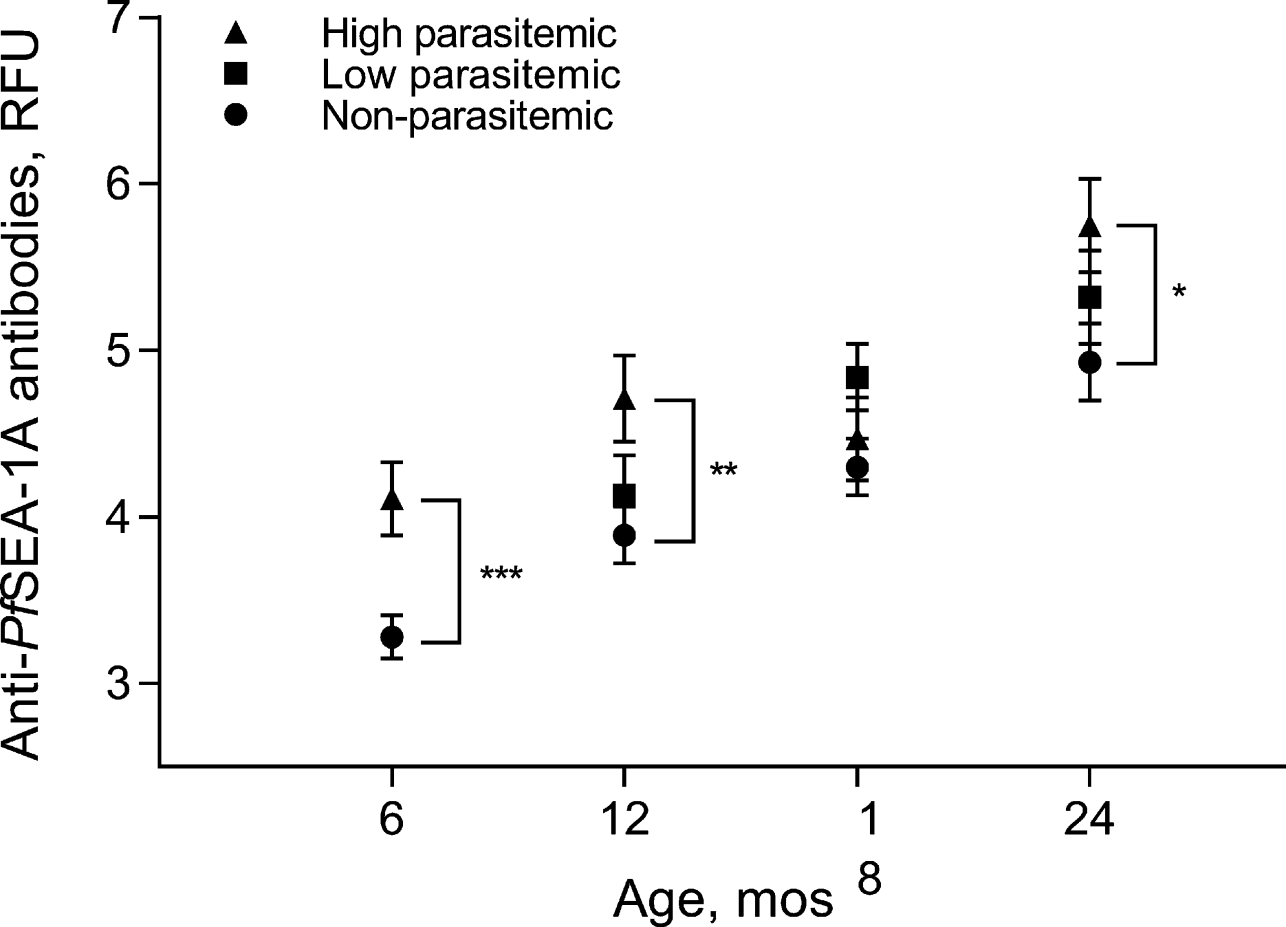
Effects of parasitemia for 6 months prior on anti-*Pf*SEA-1A antibody levels. At 6 months, infants were grouped into parasitemic and non-parasitemic, because of low prevalence of parasitemia (30.6%). At later time points, infants were grouped into tertiles of parasitemia. Anti-*Pf*SEA-1A antibody levels were natural log-transformed. Error bars represent 95% confidence intervals. ^*^P < 0.05, ^**^P < 0.01, and ^***^P < 0.001.

Infants who experienced parasitemia at least once during the first 6 months of life had significantly higher anti-*Pf*SEA-1A antibody levels at 6 and 12 months of age compared to those children who did not experience parasitemia during the first 6 months of life (Fig. 2). We next evaluated the impact of parasitemia in the preceding 6 months on anti-*Pf*SEA-1 antibody levels. Infants or children with high parasitemia in the preceding 6-month window had significantly higher anti-*Pf*SEA-1A antibody levels measured at 6, 12, and 24 months after birth compared to individuals with low or no parasitemia (Fig. 3).

**Fig. 4:**
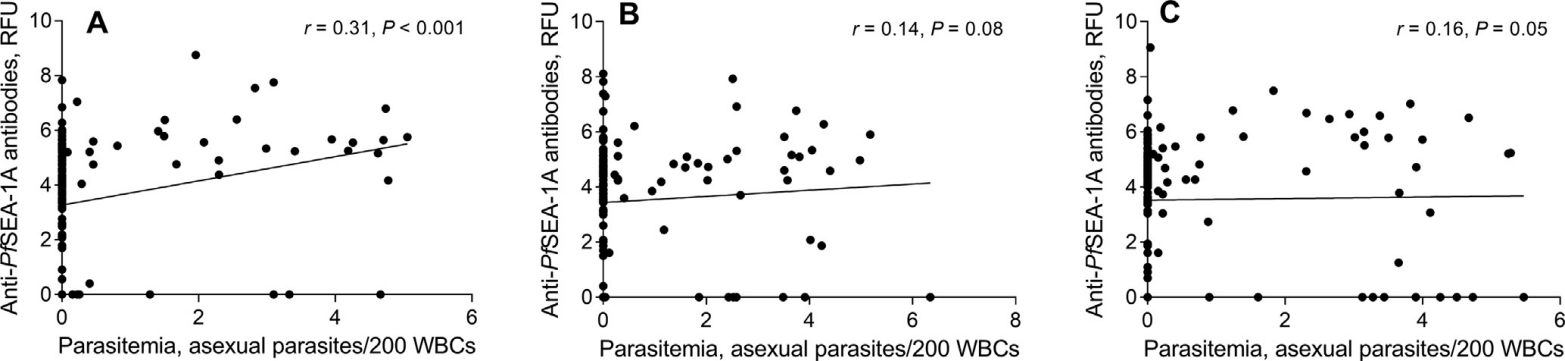
Effects of parasitemia from birth to 6 months of age on anti-*Pf*SEA-1A antibody levels at 6 months of age according to tertiles (A: low, B: middle, and C: high) of cord blood anti-*Pf*SEA-1A antibody levels. Anti-*Pf*SEA-1A antibody levels and parasitemia were natural log-transformed. Spearman correlation analysis was used to examine the correlation between parasitemia and anti-*Pf*SEA-1A antibody levels in each tertile. The interaction term between tertiles of cord blood anti-*Pf*SEA-1A antibody levels and parasitemia from birth to 6 months of age, had a significant effect on anti-*Pf*SEA-1A antibody levels (*P* value = 0.043).

To determine whether maternally-derived anti-*Pf*SEA-1 levels in cord blood altered the relationship between parasitemia and subsequent anti-*Pf*SEA-1 levels in infants and children, we fit interaction terms for these variables in our repeated measures models. Maternally-derived anti-*Pf*SEA-1A antibody levels in cord blood significantly modified the effect of parasitemia on infant anti-*Pf*SEA-1A antibody levels only at 6 months of age (Fig. 4, *P* value = 0.043). Specifically, when stratified by tertile of maternally-derived anti*-Pf*SEA-1A in cord blood, parasitemia measured from birth to 6 months of age was more strongly correlated with anti-*Pf*SEA-1A antibody levels at 6 months in the infants with low maternally-derived anti-*Pf*SEA-1A antibody levels in cord blood (Fig. 4A, *r* = 0.31, *P* value < 0.001) and this relationship was attenuated at middle (Fig. 4B, *r* = 0.14, *P* value = 0.08) and high (Fig. 4C, *r* = 0.16, *P* value = 0.05) maternally-derived anti-*Pf*SEA-1A antibody levels in cord blood. Of note, all three groups had similar anti-*Pf*SEA-1A antibody levels at six months of age (Fig. 1, Fig. 4) such that the differences in slopes across the three tertiles of maternal antibody were driven by a larger increase in anti-*Pf*SEA-1A antibodies among infants born to women with lower levels of antibody.

**Fig. 5:**
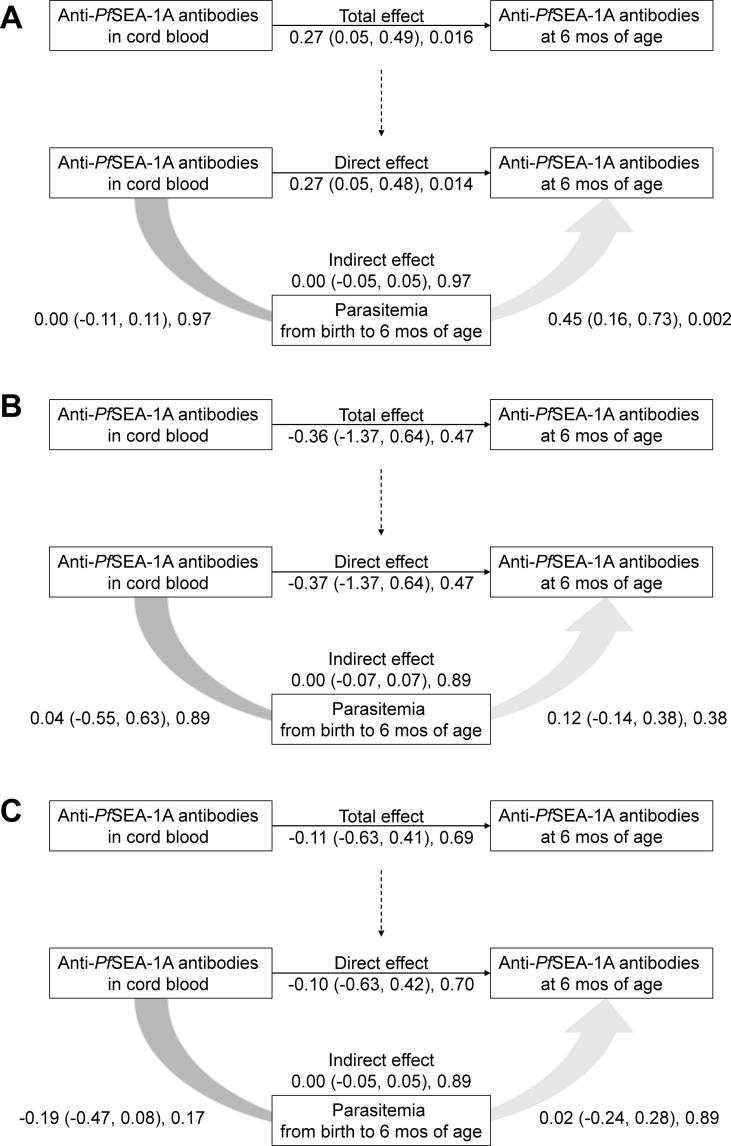
The relationship among cord blood anti-*Pf*SEA-1A antibody levels, parasitemia, and anti-*Pf*SEA-1A antibody levels at 6 months of age stratified by tertiles (A: low, B: middle, and C: high) of cord blood anti-*Pf*SEA-1A antibody levels. Anti-*Pf*SEA-1A antibody levels and parasitemia (asexual stage parasites/200 WBCs) were natural log-transformed. Numbers next to arrows indicate unstandardized coefficient (95% confidence interval), *P* value.

Consistent with this result, mediation analysis indicated that both maternally-derived anti-*Pf*SEA-1A levels in cord blood and parasitemia in the first 6 months of life had significant direct effects on offspring’s anti-*Pf*SEA-1A levels at 6 months of age in infants in the low maternal antibody tertile (Fig. 5). This was not indirectly mediated by maternally-derived anti-*Pf*SEA-1A levels in cord blood. However, neither maternally-derived anti-*Pf*SEA-1A levels in cord blood nor parasitemia in the first 6 months of life had significant effects on offspring’s anti-*Pf*SEA-1A levels at 6 months of age in infants in the middle or high maternal antibody tertiles.

## 4 Discussion

We evaluated the impact of maternally-derived anti-*Pf*SEA-1A antibody levels in cord blood and parasitemia on the development of anti-*Pf*SEA-1A antibodies among infants and children living in a malaria holoendemic region of Tanzania. Infant anti-*Pf*SEA-1A antibody levels decreased during the first 6 months of life and then increased for up to 24 months consistent with the decay of maternally-transferred anti-*Pf*SEA-1 antibodies followed by the acquisition of endogenous antibody produced in response to parasite antigen exposure.

Several studies have reported the rapid decay of transplacentally-derived antimalarial antibodies during infancy [31], [32], [35], [36]. Riley et al. [35] reported that maternally derived antimalarial antibodies to crude *P. falciparum* schizont antigen measured monthly in 143 children became undetectable in 75% of children by 22 weeks of age in a holoendemic region of southern Ghana. Using the same data, a mathematical modelling study demonstrated that antibody titers to the antigens apical membrane antigen 1, merozoite surface protein 1 and 2, and circumsporozoite protein decay in the first months of life (maternal antibody half-lives: 46, 33, 27, and 24 days, respectively) [32]. Similarly, antimalarial antibodies targeting three antigens (merozoite surface protein 3 and glutamate-rich protein [R0 and R2]) measured every 3 or 6 months in 140 children declined rapidly between one and 4 months of age in a holoendemic region of Burkina Faso [31].

Concordant with these reports, we demonstrate that anti-*Pf*SEA-1A antibody levels declined during the first 6 months of life. Importantly, maternally-derived anti-*Pf*SEA-1 antibody levels in cord blood were unrelated to anti-*Pf*SEA-1 antibody levels measured at 6 months of age, suggesting that maternal vaccination with *Pf*SEA-1 is unlikely to impact the production of endogenous anti-*Pf*SEA-1 by the infant. The low levels of anti-*Pf*SEA-1A antibody at 6 months may reflect the low incidence of malaria, possibly due to protection by maternal antibodies [5], [25], [30], immaturity of the infant immune system [37], or a combination of these factors.

Following anti-*Pf*SEA-1A antibody decay, infant anti-*Pf*SEA-1A antibody levels gradually increased over the subsequent two years of life. Parasitemia in the first 6 months of life was a strong predictor of anti-*Pf*SEA-1 levels measured at 6 and 12 months (Fig. 1). Similarly, parasitemia in the preceding 6 months was a strong predictor of anti-*Pf*SEA-1 levels measured at 6, 12 and 24 months. These results are consistent with the induction and boosting of anti-*Pf*SEA-1 antibody levels by natural exposure.

Of note, we found that maternally-derived anti-*Pf*SEA-1A antibodies in cord blood modified the development of anti-*Pf*SEA-1A antibodies by the infant in response to *P. falciparum* exposure only in the first 6 months of life. During this period, anti-*Pf*SEA-1A antibody levels were significantly related to *P. falciparum* exposure in infants born with low, but not higher, maternally-derived anti-*Pf*SEA-1A antibody levels in cord blood (Fig. 4). Given all three groups ultimately had similar anti-*Pf*SEA-1A antibody levels at six months of age (Fig. 1, Fig. 4), the difference in the correlations between natural exposure (parasitemia) and infant antibody levels across the tertiles of cord blood antibody levels were driven by a larger increase in anti-*Pf*SEA-1A antibodies among infants born to women with lower levels of antibody, rather than a significant decline in antibody levels among infants born to mothers with higher anti-*Pf*SEA-1A antibody levels. This suggests that *Pf*SEA-1 may be more immunogenic in infants with low maternally-derived anti-*Pf*SEA-1A antibody levels [30], possibly due to antigen clearance in infants with higher maternal antibody levels. Maternally-derived anti-*Pf*SEA-1A antibody levels, however, do not interfere with the maintenance or development of antibody levels at six months of age among infants with higher maternal antibody levels. Consistent with this interpretation, mediation analysis indicated that maternally-derived anti-*Pf*SEA-1A antibodies in cord blood did not influence anti-*Pf*SEA-1A antibody levels in response to *P. falciparum* exposure (indirect effect) in infants born with any level of maternal antibody.

To our knowledge, this is the first study to investigate the dynamics of maternally-transferred and naturally acquired antibodies to *Pf*SEA-1, a promising vaccine candidate antigen for controlling *P. falciparum* infection. Nevertheless, our study has several limitations. First, we did not directly determine the relative proportion of maternally-transferred antibodies versus endogenous, naturally acquired antibodies in infants and children. Instead, we relied on the decline followed by rise in concentration of anti-*Pf*SEA-1 antibody to infer their source. In addition, we only evaluated anti-*Pf*SEA-1 IgG response. While some studies report minimal detection of *Pf*-specific IgM in cord blood [19], [38], [39], others report detecting *Pf*-specific IgM in nearly 30% of African neonates [40], [41], [42], [43]. Several studies have found *Pf*-specific cord blood IgM to be altered in response to *in utero* malaria exposure [41], [43], but how these alterations may impact early childhood infection remains unknown. Further, recent published data suggests that placental malaria may result in maternal microchimerism making it difficult to classify IgM antibodies as truly fetal as they may be maternal IgM due to *in utero* exposure [44]. Second, we did not measure anti-*Pf*SEA-1A antibody levels at additional time points between birth and 6 months of age, and thus, we cannot define at what point these young infants reached their antibody nadir. Third, the availability of serum samples resulted in loss to follow-up such that 18.1% (n = 117) of the total study population were not followed after 6 months. However, their maternally-derived anti-*Pf*SEA-1A antibody levels in cord blood and parasitemia during the first 6 months were not different from the children followed past 6 months (Wilcoxon rank-sum test, *P* values > 0.7). Fourth, our study does not take into consideration the effect sub-patent parasitemia may have on antibody production because parasitemia was determined by microscopy and not polymerase chain reaction (PCR). Despite the limit of detection for the method used, we do detect a significant difference in anti-*Pf*SEA-1A antibody levels between infants designated as non-parasitemic and parasitemic at 6 and 12 months. PCR determination could potentially expose differences in antibody production at later time points (18 and 24 months) and should be considered in future studies.

In conclusion, our study demonstrates that natural exposure to *P. falciparum* increases the levels of naturally acquired anti-*Pf*SEA-1A antibodies in infants and young children living in a holoendemic setting. Importantly, maternally derived anti-*Pf*SEA-1A antibodies did not interfere with the development of endogenous anti-*Pf*SEA-1 responses in infants. This observation, taken together with our recently published data demonstrating that maternal vaccination with *Pf*SEA-1 conferred protection upon *P. berghei* malaria challenge to the pups [25], diminishes the concern that the use of malaria vaccines before or during pregnancy might interfere with acquisition of antibodies by their offspring.

## 5 Financial support

This work was supported by grants from the US National Institutes of Health [grant R01-AI52059] and the Bill & Melinda Gates Foundation [grant 1364] to PED, the Intramural Research Program of the NIAID-NIH, and by grants from the US National Institutes of Health [grants R01-AI076353, R01 AI110699-01] to JDK. CEN was supported by the US National Institutes of Health [grant T32-DA013911] and The Thrasher Research Fund. JFF and SP were supported by US National Institutes of Health [grant P20GM104317-01] and JFF by US National Institutes of Health [grant K24 AI112964].

## Declaration of Competing Interest

All authors: No potential conflicts of interest.
